# Cardiac Implantable Electronic Device Lead Perforation: A 25-Year Single-Center Experience

**DOI:** 10.3390/jcm15072705

**Published:** 2026-04-02

**Authors:** Sameer Al-Maisary, Matthias Karck, Mario Jesus Guzman-Ruvalcaba, Rawa Arif, Gabriele Romano

**Affiliations:** 1 Department of Cardiothoracic Surgery, Saarland University Hospital, 66421 Homburg, Germany; 2 Department of Cardiac Surgery, Heidelberg University Hospital, 69120 Heidelberg, Germany

**Keywords:** cardiac implantable electronic device, lead perforation, computed tomography

## Abstract

**Background:** Cardiac implantable electronic device (CIED) lead perforation is a rare but potentially catastrophic complication. As global device implantations increase, understanding the clinical spectrum and optimal management of this complication is essential. This study characterizes the clinical presentation, diagnostic strategies, and outcomes of lead perforation over a 25-year period. **Methods:** A retrospective analysis was conducted on 32 patients diagnosed with CIED lead perforation between 2000 and 2025 at a high-volume center. Perforations were classified by timing: acute (<24 h), subacute (1–30 days), and chronic (>30 days). Data included demographics, comorbidities, imaging modalities, and procedural interventions. **Results:** The mean patient age was 76.0 ± 11.7 years, with a mean body mass index (BMI) of 25.5 ± 3.4 kg/m^2^. Subacute presentation was the most frequent (59.3%, n = 19), followed by acute (28.1%, n = 9) and chronic (12.5%, n = 4) cases. The right ventricle was the primary site of perforation (90.6%). While chest X-rays served as an initial screening tool in 62.5% of cases, diagnosis relied on multimodal imaging, with Computed Tomography (CT) providing definitive confirmation in 31.3% of the cohort, particularly when lead parameters remained stable. Management was risk-stratified based on hemodynamic status. The majority of patients (71.9%, n = 23) underwent successful transvenous lead removal via simple traction. However, 25% (n = 8) presented with hemodynamic instability, and 21.9% (n = 7) suffered from cardiac tamponade. These high-risk cases required surgical intervention, including sternotomy (n = 4), thoracotomy (n = 2), or pericardiotomy (n = 3). Notably, 62.5% of hemodynamically unstable patients were on oral anticoagulants. All patients survived to discharge, with no in-hospital mortality. The median length of hospital stay was 3 days. **Conclusions:** CIED lead perforation often presents subacutely with subtle clinical signs. CT imaging has emerged as the gold standard for definitive diagnosis. While percutaneous transvenous removal is safe and effective for stable patients, immediate surgical backup is vital, as patients—particularly those on anticoagulation—can deteriorate rapidly.

## 1. Introduction

As the clinical application of cardiac implantable electronic devices follows updated appropriate use criteria [1], lead perforation remains a rare but potentially lethal complication [2]. Historically, the global incidence rate of this complication has been reported between 0.1% and 0.8% [3]. However, recent meta-analyses suggest the real-world incidence may be higher, potentially reaching 1% to 2%, especially when asymptomatic cases are detected on routine imaging [4]. This event occurs when a pacing lead traverses the myocardium, primarily affecting either the right atrium [5] or the right ventricle [6].

Several patient-specific characteristics increase the risk of perforation. Advanced age is identified as a primary risk factor due to decreased myocardial tensile strength [4]. Furthermore, a low body mass index is frequently cited as a predictor for this complication [7]. Perforations are categorized by their timing of onset, ranging from acute cases during or immediately after the implant procedure to subacute presentations [6], and even chronic or delayed cases occurring years after the initial procedure [8]. Historically, acute perforations were more common, likely due to older, stiffer lead designs [9]. Today, vigilance beyond the immediate perioperative period is crucial, as symptoms like diaphragmatic stimulation can be delayed [9,10].

The clinical spectrum of perforation is exceptionally broad, posing a significant diagnostic challenge in emergency settings [11]. Patients may present with acute chest pain [12] or be identified incidentally during investigations for lead dislodgement [13]. In severe instances, the lead can migrate into adjacent anatomical structures, leading to pneumothorax [14] or life-threatening circulatory collapse from arterial injury [15]. Accurate diagnosis relies heavily on multimodal imaging. While chest X-rays are frequently utilized as an initial screening tool, they lack the diagnostic sensitivity provided by more advanced modalities. Contemporary studies advocate for Computed Tomography (CT) as the gold standard, offering precise, high-resolution visualization of the lead tip relative to the pericardium and surrounding structures [10,16].

Management strategies are dictated by hemodynamic stability and the extent of injury. The evolution of these strategies has progressively shifted toward less invasive approaches. While some cases are managed conservatively with simple transvenous traction as a safe and effective first-line therapy for stable patients [16,17], others require invasive surgical repair, such as a thoracotomy, to definitively resolve ventricular perforation and secure hemostasis [18]. Careful multidisciplinary planning and the immediate availability of surgical backup are essential components of safe lead extraction protocols. Understanding these diverse clinical variations is vital for optimizing outcomes in pacing therapy.

## 2. Methods

### 2.1. Study Design and Ethical Oversight

This study utilized a single-center, descriptive retrospective analysis to investigate the clinical characteristics and management of cardiac implantable electronic device (CIED) lead perforations. The study period spanned 25 years, from January 2000 to December 2025, capturing a broad evolution in both pacing technology and surgical techniques. All research activities were conducted at a high-volume tertiary care center, with patients either referred from external regional hospitals for specialized management or identified within the facility’s own cardiology clinic.

### 2.2. Patient Population and Data Extraction

A total of 32 patients were identified who met the inclusion criteria of a confirmed CIED lead perforation. To ensure data integrity, researchers performed a systematic extraction of information from the institutional electronic medical records. This data was then organized into a standardized, secure database for analysis.

The data collection focused on five primary domains:

1. Demographics: Basic patient identifiers, including age, biological sex, height, and weight, to calculate Body Mass Index (BMI).
2. Clinical Status: Pre-procedural Left Ventricular Ejection Fraction (LVEF), assessed via transthoracic or transesophageal echocardiography (TTE/TEE), and functional status according to the NYHA classification.
3. Comorbidities: Documentation of preexisting conditions such as Arterial Hypertension (HTN), Diabetes Mellitus, and coronary heart disease (CHD) based on clinical diagnosis or active medication usage.
4. Device Specifications: Records of the type of CIED implanted (e.g., dual-chamber pacemakers) and the specific anatomical site of the perforating lead (Atrium vs. Ventricle).
5. Anticoagulation Status: Identification of patients on oral anticoagulants or steroids at the time of the complication.

### 2.3. Definitions of Perforation Acuity

For this analysis, lead perforations were categorized by the timing of clinical presentation or diagnostic confirmation relative to the date of initial implantation. These categories were defined as follows:

- Acute: Perforation occurring during the implant procedure or within the first 24 h.
- Sub-acute: Presentation occurring between 1 and 30 days post-implantation.
- Chronic: Perforation diagnosed more than 30 days after the initial procedure.

### 2.4. Diagnostic Protocol and Imaging

The center utilized a multimodal imaging approach to diagnose and risk-stratify lead perforations. The diagnostic pathway typically followed a tiered progression:

- Initial Screening: Chest X-rays were used to define the general location of the lead tip and identify gross displacement.
- Hemodynamic Assessment: Echocardiography (TTE/TEE) was employed to assess for the presence of pericardial effusion or the development of life-threatening cardiac tamponade.
- Definitive Confirmation: Computed Tomography (CT) served as the gold standard, providing high-resolution visualization to confirm the exact position of the lead tip relative to the pericardium and surrounding thoracic structures.
- Device Interrogation: Electronic assessment of sensing, pacing thresholds, and lead impedance was performed to identify subtle changes indicative of a lead crossing the myocardial wall.

### 2.5. Procedural Management and Surgical Intervention

Safety was prioritized by performing all lead-related interventions in an operating theater under general anesthesia. Continuous invasive monitoring of blood pressure and vital signs was mandatory for all patients.

Management was strictly stratified by hemodynamic stability:

1. Stable Patients: For those without signs of tamponade, simple transvenous traction was the preferred method for lead removal. In this cohort, specialized tools like laser sheaths were not required.
2. Unstable Patients: In cases involving hemodynamic instability or significant pericardial tamponade, immediate surgical intervention was initiated. Pericardiotomy was performed via various approaches, depending on clinical exigency, including sternotomy, thoracotomy, inferior pericardiotomy, or pericardiocentesis.
3. Monitoring: Transesophageal echocardiography (TEE) was utilized throughout the extraction process to monitor for emergent pleural or pericardial effusions.

### 2.6. Statistical Analysis

Statistical processing was conducted to describe the central tendencies and distributions of the cohort. Continuous variables were presented as mean ± standard deviation (SD) for normally distributed data. To account for clinical outliers in hospital length of stay, these data were reported as median values with interquartile ranges (IQRs). Categorical variables (such as sex, presence of comorbidities, and type of intervention) were summarized using frequencies and percentages.

## 3. Results

### 3.1. Patient Demographics and Baseline Characteristics

A comprehensive retrospective analysis was conducted on a carefully defined cohort of 32 patients who presented with confirmed cardiac implantable electronic device (CIED) lead perforations over the 25-year study period spanning from 2000 to 2025. The patient population primarily consisted of an older demographic, characterized by a substantial burden of preexisting systemic and cardiovascular vulnerabilities. The mean age of the patients at the time of clinical presentation was calculated to be 76.0 ± 11.7 years. The distribution of biological sex within this cohort was perfectly symmetrical, with female patients accounting for exactly half (50%, n = 16) of the total study population (Table 1).

Body composition analysis revealed a mean Body Mass Index (BMI) of 25.5 ± 3.4 kg/m^2^ across the 32 patients. However, this central tendency encompassed a broad and diverse clinical spectrum, with individual BMI values ranging from severely cachectic (17.8 kg/m^2^) to obese (34.0 kg/m^2^). Prior to or at the exact time of the perforation complication, the vast majority of the patient cohort was categorized as possessing a New York Heart Association (NYHA) Class I functional status. Furthermore, the mean left ventricular ejection fraction (LVEF), as assessed through comprehensive echocardiographic imaging, was relatively preserved across the population at 53.7 ± 6.4%.

### 3.2. Comorbidity Profile and Medical Management

The baseline comorbidity profile highlighted a highly significant prevalence of cardiovascular risk factors that likely contributed to the complexity of patient management. Arterial hypertension (HTN) was the most frequently documented underlying comorbidity, affecting a substantial 68.7% (n = 22) of the patients. Diabetes Mellitus was also highly prevalent, diagnosed in 34.3% (n = 11) of the study group. Furthermore, established coronary heart disease (CHD) was documented in 37.5% (n = 12) of the cohort. Renal insufficiency was noted in a smaller, yet clinically relevant subset, comprising 12.5% (n = 4) of the patients.

Concurrent medical management included the systemic use of steroids, which was recorded in only a single patient (3.1%). Atrial fibrillation was present in exactly one-quarter of the cohort (25%, n = 8) at the time they presented with the perforation. Crucially, regarding the complication risk profile and subsequent bleeding management, more than a third of the cohort—37.5% (n = 12)—were actively receiving oral anticoagulant therapy when the perforation event occurred.

### 3.3. Device Specifications and Perforation Timing

Regarding the technical specifications of the implanted pacing systems, the overwhelming majority of index procedures involved the placement of standard dual-chamber pacemakers, accounting for 24 distinct cases. Notably, among this cohort of 32 patients, no perforations involving complex devices, such as Implantable Cardioverter-Defibrillators (ICD) or Cardiac Resynchronization Therapy (CRT) systems, were recorded (Table 2).

The mean chronological age of the perforating leads—defined as the specific time elapsed from the initial surgical implantation procedure to the definitive diagnosis of perforation—was calculated to be 16.7 days. When stratifying the perforations by their temporal onset, a subacute presentation (defined as occurring between 1 and 30 days post-implantation) was by far the most dominant, representing 59.3% (n = 19) of the entire cohort. Acute perforations, which occurred within the immediate 24-h window post-implantation, comprised 28.1% (n = 9) of the cases. Chronic perforations, which are delayed complications manifesting more than 30 days post-procedure, were the least common clinical presentation, observed in 12.5% (n = 4) of the patient population.

Anatomically, the perforations exhibited an overwhelming predilection for the right ventricular anatomy. Leads positioned within the Right Ventricle (RV) accounted for the vast majority of perforation events, representing 90.6% (n = 29) of all documented cases. By stark contrast, perforations originating from leads implanted in the Right Atrium (RA) were markedly less frequent, occurring in only 9.4% (n = 3) of the cohort.

### 3.4. Clinical Presentation and Diagnostic Modalities

Upon presentation to the clinical facility, the hemodynamic status of the patients was sharply divided, dictating subsequent care pathways. The majority of the cohort (24 patients) remained entirely hemodynamically stable at the time of their diagnostic evaluation. Among these 24 stable individuals, nearly all (n = 23) demonstrated clear echocardiographic evidence of a pericardial effusion. Conversely, a critical and high-risk subset of 8 patients (25.0%) presented with severe hemodynamic instability. This acute instability was primarily driven by the active development of cardiac tamponade, which was definitively diagnosed in 7 patients (21.9% of the total cohort).

The precise identification and structural confirmation of the lead perforations necessitated a rigorous, multimodal imaging methodology. A standard chest X-ray (XR) served as the primary screening tool for a significant portion of the cohort (n = 20). However, this initial radiographic screening frequently lacked the required anatomical specificity and mandated secondary, high-fidelity imaging modalities to achieve definitive diagnostic confirmation. Computed Tomography (CT) was heavily utilized and proved highly instrumental in establishing a definitive diagnosis for a large segment of patients (n = 10) by providing high-resolution visualization of the trans-myocardial lead trajectory. Furthermore, echocardiography was concurrently employed as an essential supplementary tool in 6 cases, specifically utilized to evaluate, monitor, and quantify the hemodynamic impact of the resulting pericardial effusions.

### 3.5. Surgical Management and Intervention Strategies

**Figure 1:**
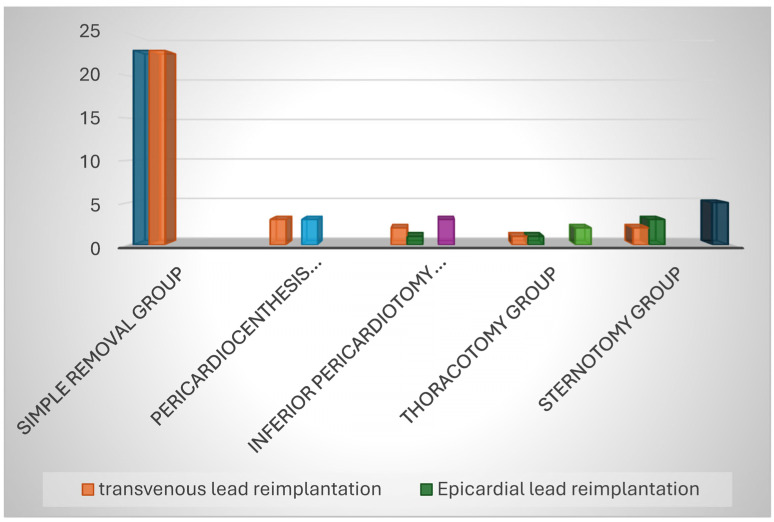
Operative Management.

The chosen operative and management strategies were strictly dictated by the initial hemodynamic presentation. For the 24 hemodynamically stable patients, conservative, percutaneous approaches were strongly prioritized. Of this stable subgroup, 20 patients underwent straightforward, successful simple removal of the offending perforating lead via transvenous traction. Across the entire cohort, regardless of initial presentation, a total of 23 patients (71.9%) were ultimately managed through simple transvenous lead removal (Table 3) (Figure 1).

**Figure 2:**
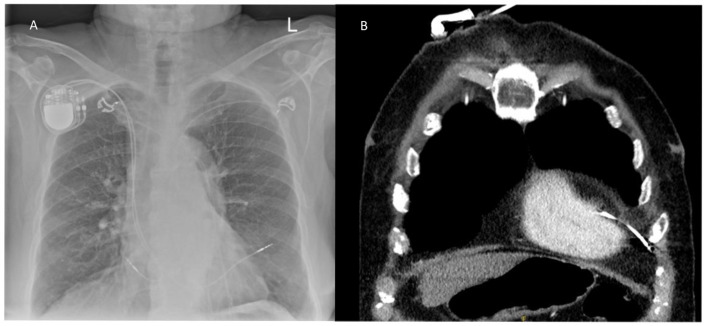
(**A**) Shows chest X-Ray with a right ventricular lead perforation, (**B**) Shows a computer tomography with right ventricular lead perforation.

Surgical intervention, while significantly less frequent overall, was absolutely necessary for specific, high-risk clinical scenarios. Historical surgical data within the 25-year span revealed that 4 otherwise stable patients underwent more invasive surgical procedures early in the study’s timeline (Figure 2). Specifically, three hemodynamically stable patients underwent a full surgical sternotomy (occurring historically in the years 2004, 2007, and 2008), and one stable patient underwent a left lateral thoracotomy in 2006.

The management of the 8 hemodynamically unstable patients necessitated immediate, life-saving invasive measures. Within this critical subgroup, the targeted surgical interventions varied based on patient anatomy and severity: three patients required an inferior pericardiotomy to relieve tamponade, one patient was managed via a left lateral thoracotomy, and one patient required an emergent full sternotomy. An additional three unstable patients were successfully managed through emergency pericardiocentesis coupled with simple transvenous lead removal. Overall, reviewing the total cohort interventions, surgical sternotomy was performed in 4 cases (12.5%), surgical thoracotomy in 2 cases (6.2%), and pericardiocentesis and pericardiotomy were each utilized in 3 cases (9.4% each).

A highly notable clinical correlation was directly observed within the hemodynamically unstable subgroup: 5 out of the 8 critically unstable patients were actively receiving oral anticoagulant therapy at the precise time of their rapid clinical decompensation. Following successful extraction of the perforated leads, the strategy for re-establishing permanent cardiac pacing varied according to surgical access. Five patients in the cohort required the direct surgical placement of epicardial pacemaker leads. This specific epicardial subgroup included 3 patients who had undergone a sternotomy, 1 patient who received a thoracotomy, and 1 patient who underwent an inferior pericardiotomy. However, the vast majority of the overall cohort (n = 27) were safely and simultaneously re-implanted with new transvenous permanent pacing leads during their index hospital admission.

### 3.6. Hospital Course and Clinical Outcomes

Despite the highly advanced average age of the cohort, the significant burden of preexisting cardiovascular comorbidities, and the inherent, life-threatening risks intimately associated with both cardiac perforation and emergency surgical lead extractions, the overall clinical outcomes were exceptional. The hospital stay length for the entire study population was highly efficient, with a median of precisely 3 days. Taking clinical outliers regarding recovery into account, the hospital stay duration yielded an interquartile range of 3 ± 5.8 days. Most notably, careful risk-stratification protocols, the systematic use of advanced multimodal imaging, and the immediate, uninterrupted availability of surgical interventions culminated in complete survival for the entire cohort. Zero instances of in-hospital mortality (0.0%) were recorded across all 32 patients throughout the entire 25-year study duration.

## 4. Discussion

In this 25-year retrospective analysis, we evaluated 32 cases of cardiac implantable electronic device (CIED) lead perforation. Our findings highlight that while lead perforation remains a rare but potentially lethal complication [2], management in a high-volume center yields excellent clinical outcomes despite an aging patient population with significant cardiovascular comorbidities.

The incidence of lead perforation in our series aligns with global rates, typically reported between 0.1% and 0.8% [3]. However, the recent literature suggests the real-world incidence may be higher—potentially reaching 1% to 2%—when subclinical or asymptomatic cases are identified through routine imaging [4,19]. The demographic profile of our cohort, with a mean age of 76 years, reinforces the understanding that advanced age is a primary risk factor for myocardial wall fragility [4,17]. Interestingly, while a low body mass index (BMI) is frequently cited as a predictor for perforation [7], our cohort maintained a mean BMI of 25.5 kg/m^2^, suggesting that risk is not limited solely to cachectic patients.

We observed a distinct predominance of subacute perforations (59.3%), occurring between 1 and 30 days post-implant. This shift from historical trends, where acute events were more frequent, likely reflects improvements in lead design and implant techniques [9]. However, this high subacute rate emphasizes the critical need for vigilance beyond the immediate 24-h perioperative window, as symptoms like atypical chest pain or diaphragmatic stimulation can be delayed [9,10].

Our data strongly support the necessity of multimodal imaging for accurate diagnosis. While chest X-ray served as the primary screening tool in 50% of our cases, its sensitivity is notably limited. Computed Tomography (CT) has emerged as the “gold standard” in this context, providing high-resolution visualization of the lead tip relative to the pericardium [10,20]. In our experience, CT was instrumental in confirming the diagnosis for 34.4% of patients, particularly when electrical lead parameters remained deceptively stable.

Management strategies for lead perforation have transitioned toward less-invasive, percutaneous approaches [16]. In our study, 71.9% of patients were successfully managed with simple transvenous lead removal and repositioning. This confirms that traction is a safe first-line therapy for hemodynamically stable patients [16,17]. Conversely, hemodynamic instability—present in 25% of our patients—remains a definitive indication for surgical intervention [21,22]. We found that patients on oral anticoagulants were particularly susceptible to rapid deterioration and cardiac tamponade, necessitating more aggressive measures such as sternotomy or thoracotomy [22].

The 0% in-hospital mortality recorded in our series demonstrates the efficacy of a multidisciplinary “Heart Team” approach. While percutaneous removal is preferred, immediate surgical backup is essential to mitigate the risk of catastrophic hemorrhage during lead extraction [17,21]. By combining rapid diagnostic CT with risk-stratified management, clinical outcomes can be optimized even in the most complex presentations of CIED lead perforation [17,21].

## 5. Conclusions

In summary, cardiac implantable electronic device (CIED) lead perforation is a rare but potentially catastrophic complication that demands a high index of clinical suspicion. This 25-year study of 32 patients reveals that the majority of perforations occur subacutely (59.3%) and primarily affect the right ventricle (90.6%). While chest X-rays serve as an initial screening tool, Computed Tomography (CT) has emerged as the diagnostic gold standard for definitive lead tip localization.

This study of 32 patients reveals several critical clinical applications for managing cardiac implantable electronic device (CIED) lead perforations. First, institutions should update postoperative monitoring protocols to reflect that 59.3% of perforations occur subacutely between 1 and 30 days, requiring vigilance well beyond the initial 24-h perioperative window. Second, the diagnostic pathway should be standardized to prioritize Computed Tomography (CT) as the gold standard for definitive confirmation, as it provides high-resolution visualization even when electronic lead parameters appear deceptively stable. Third, management must be strictly risk-stratified based on hemodynamic stability. Meanwhile, simple transvenous traction is a safe first-line therapy for the 71.9% of stable patients, those on oral anticoagulants require heightened alert status due to their susceptibility to rapid clinical decompensation and cardiac tamponade. Finally, this research mandates specific institutional safety infrastructure, asserting that all lead extractions must be performed in an operating theater under general anesthesia with a multidisciplinary “Heart Team” and immediate surgical backup available to manage life-threatening complications.

### Study Limitations

This is a retrospective Design, which is subject to selection bias and reliance on historical records. The small cohort (n = 32) limits statistical power. Results from a single center may not generalize to smaller hospitals. Also, the 25-year span introduces confounding variables in lead design and imaging quality. The lack of long-term follow-up is also a study limitation.

## Figures and Tables

**Table 1:** Patient Demographics.

Characteristics	N = 32
Age (years)	76 ± 11.7
Female, n (%)	16 (50)
BMI	25.5 ± 3.4
EF (%)	53.7 ± 6.4
DM, n (%)	11 (34.3)
HTN, n (%)	22 (68.7)
Renal insufficiency, n (%)	4 (12.5)
Coronary heart disease, n (%)	12 (37.5)
Steroids, n (%)	1 (3.1)
Atrial fibrillation, n (%)	8 (25)
Oral Anticoagulation, n (%)	12 (37.5)

**Table 2:** Perforation Characteristics.

Category	N (%)
**Time to Perforation**	
Acute (<24 h)	9 (28.1%)
Subacute (1–30 days)	19 (59.3%)
Chronic (>30 days)	4 (12.5%)
**Perforating Lead Location**	
Right Ventricle (RV)	29 (90.6%)
Right Atrium (RA)	3 (9.4%)

**Table 3:** Management and Clinical Outcomes.

Outcome/Procedure	Value
**Clinical Presentation**	
Hemodynamic Instability	8 (25.0%)
Cardiac Tamponade	7 (21.9%)
**Lead Management Strategy**	
Simple Lead Removal (Transvenous)	23 (71.9%)
Surgical Sternotomy	4 (12.5%)
Surgical Thoracotomy	2 (6.2%)
**Interventions**	
Pericardiocentesis	3 (9.4%)
Pericardiotomy	3 (9.4%)
**Hospital Course**	
Length of Stay (days, IQR *)	3
In-hospital Mortality	0 (0.0%)

* IQR: Interquartile Range.

## Data Availability

Data is contained within the Supplementary Materials.

## Supplementary Materials

The following supporting information can be downloaded at https://www.mdpi.com/article/10.3390/jcm15072705/s1. Table S1: Data set.

## References

[1] Ferrick A.M., Raj S.R., Deneke T., Kojodjojo P., Lopez-Cabanillas N., Abe H., Boveda S., Chew D.S., Choi J.I., Dagres N. (2023). 2023 HRS/EHRA/APHRS/LAHRS Expert Consensus Statement on Practical Management of the Remote Device Clinic. Europace.

[2] Vanezis A.P., Prasad R., Andrews R. (2017). Pacemaker leads and cardiac perforation. JRSM Open..

[3] Migliore F., Zorzi A., Bertaglia E., Leoni L., Siciliano M., De Lazzari M., Ignatiuk B., Veronese M., Verlato R., Tarantini G. (2014). Incidence, management, and prevention of right ventricular perforation by pacemaker and implantable cardioverter defibrillator leads. Pacing Clin. Electrophysiol..

[4] Awashra A., AbuBaha M., Salameh H., Jallad H., Milhem A., Zahran A., Badwan A., Milhem F., Shubietah A. (2025). Cardiac lead perforation: Mechanisms, detection, and therapeutic approaches. Heart Rhythm O2.

[5] Vegas Pinto R., Beryanaki M.Z., Torralvo V.P. (2022). Atrial perforation by pacemaker lead. Rev. Esp. Cardiol. (Engl. Ed.).

[6] Allouche E., Chargui S., Fathi M., Bezdah L. (2021). Subacute right ventricle perforation: A pacemaker lead complication. BMJ Case Rep..

[7] Cano Ó., Andrés A., Alonso P., Osca J., Sancho-Tello M.J., Olagüe J., Martínez-Dolz L. (2017). Incidence and predictors of clinically relevant cardiac perforation associated with systematic implantation of active-fixation pacing and defibrillation leads: A single-centre experience with over 3800 implanted leads. Europace.

[8] Yamamoto A., Takahashi S. (2022). Delayed right ventricular lead perforation by a pacemaker lead 2-year post-implantation. Clin. Case Rep..

[9] Ahmed A., Shokr M., Lieberman R. (2017). Subacute Right Ventricular Perforation by Pacemaker Lead Causing Left-Sided Hemothorax and Epicardial Hematoma. Case Rep Cardiol..

[10] Rajkumar C.A., Claridge S., Jackson T., Behar J., Johnson J., Sohal M., Amraoui S., Nair A., Preston R., Gill J. (2017). Diagnosis and management of iatrogenic cardiac perforation caused by pacemaker and defibrillator leads. Europace.

[11] Kumar P., Skrabal J., Frasure S.E., Pourmand A. (2022). Pacemaker lead related myocardial perforation. Am. J. Emerg. Med..

[12] Cañizares-Otero M.C., Danckers M. (2021). Pacemaker Lead Migration and Ventricular Perforation in a Patient Presenting with Chest Pain. Clin. Pract. Cases Emerg. Med..

[13] Menexi C., ElRefai M. (2024). Pacemaker-Lead Dislodgement and Cardiac Perforation. N. Engl. J. Med..

[14] Iwata S., Hirose A., Furui I., Matsumoto T., Ozaki M., Nagasaka Y. (2021). Right ventricular perforation, pneumothorax, and a pneumatocele by a pacemaker lead: A case report. JA Clin. Rep..

[15] Kumar R., Kumar J., Ullah I., Edroos S.A., Matiullah S. (2022). Lead Perforation Into the Left Internal Mammary Artery Causing Circulatory Collapse After Pacemaker Insertion. JACC Case Rep..

[16] Döring M., Müssigbrodt A., Ebert M., Bode K., Lucas J., Dagres N., Hindricks G., Richter S. (2020). Transvenous revision of leads with cardiac perforation following device implantation-Safety, outcome, and complications. Pacing Clin. Electrophysiol..

[17] Waddingham P.H., Elliott J., Bates A., Bilham J., Muthumala A., Honarbakhsh S., Ullah W., Hunter R.J., Lambiase P.D., Lane R.E. (2022). Iatrogenic cardiac perforation due to pacemaker and defibrillator leads: A contemporary multicentre experience. Europace.

[18] Uemura H., Yajima S., Sekiya N., Yamazaki S., Satoh A., Tanaka H., Yamamura M., Sakaguchi T. (2021). Repair of pacemaker lead-induced right ventricular perforation via a left mini-thoracotomy. J. Cardiol. Cases.

[19] Dar M.I., Hafeez I., Tahir S.M., Sheikh J.M., Ganie F.A., Bilal S., Lone A.A., Rather H.A. (2025). Pacemaker lead perforations: A five-year study from a high-volume center in India. Egypt Heart J..

[20] Henrikson C.A., Leng C.T., Yuh D.D., Brinker J.A. (2006). Computed tomography to assess possible cardiac lead perforation. Pacing Clin. Electrophysiol..

[21] Younis A., Awashra A., Matteo M., Hussein A.A., Demian J., Santangeli P., Callahan T., Martin D.O., Mdaihly M., Nakhla S. (2025). Management of Transvenous Leads in Patients with Iatrogenic Lead Perforation. J. Cardiovasc. Electrophysiol..

[22] Liu Y., Liu H., Zhu B., Li H., Cui N., Ye F., Gao J., Zhang X., Wu Y. (2025). Study on the Identification and Treatment Strategy of Cardiac Perforation Caused by Pacing Lead: A Systematic Review and Meta-Analysis. Cardiology.

